# The Need to Work Arm in Arm: Calling for Collaboration in Delivering Neuroprosthetic Limb Replacements

**DOI:** 10.3389/fnbot.2021.711028

**Published:** 2021-07-21

**Authors:** Alison M. Karczewski, Aaron M. Dingle, Samuel O. Poore

**Affiliations:** Division of Plastic Surgery, Department of Surgery, University of Wisconsin–Madison, Madison, WI, United States

**Keywords:** neuroprosthetic, amputation, prosthesis, peripheral nerve interface, neuroprosthetic interfacing, sensory motor function, clinical translation, human machine collaboration

## Abstract

Over the last few decades there has been a push to enhance the use of advanced prosthetics within the fields of biomedical engineering, neuroscience, and surgery. Through the development of peripheral neural interfaces and invasive electrodes, an individual's own nervous system can be used to control a prosthesis. With novel improvements in neural recording and signal decoding, this intimate communication has paved the way for bidirectional and intuitive control of prostheses. While various collaborations between engineers and surgeons have led to considerable success with motor control and pain management, it has been significantly more challenging to restore sensation. Many of the existing peripheral neural interfaces have demonstrated success in one of these modalities; however, none are currently able to fully restore limb function. Though this is in part due to the complexity of the human somatosensory system and stability of bioelectronics, the fragmentary and as-yet uncoordinated nature of the neuroprosthetic industry further complicates this advancement. In this review, we provide a comprehensive overview of the current field of neuroprosthetics and explore potential strategies to address its unique challenges. These include exploration of electrodes, surgical techniques, control methods, and prosthetic technology. Additionally, we propose a new approach to optimizing prosthetic limb function and facilitating clinical application by capitalizing on available resources. It is incumbent upon academia and industry to encourage collaboration and utilization of different peripheral neural interfaces in combination with each other to create versatile limbs that not only improve function but quality of life. Despite the rapidly evolving technology, if the field continues to work in divided “silos,” we will delay achieving the critical, valuable outcome: creating a prosthetic limb that is right for the patient and positively affects their life.

## Introduction

Limb amputation is a transformative, debilitating life event that has the ability to drastically impair one's quality of life. Consequential psychosocial stressors associated with limb amputation can affect careers, personal relationships, and threaten an individual's sense of self (Resnik et al., 2011; Sinha et al., 2011). There are ~2.2 million people living with limb loss in the United States (Ziegler-Graham et al., 2008). As that number continues to climb, efforts to improve amputation-related morbidity have led to collaborations between experts in the fields of medicine and engineering and the development of innovative rehabilitation strategies. Advancements in biomechanics and prosthetic technology have focused on improving functionality and restoring a sense of embodiment in those who have undergone amputations. Similarly, new surgical techniques have been created to further optimize prosthetic use and alleviate chronic postamputation pain (Mioton and Dumanian, 2018). In contrast with these rapid developments, the surgical approach to limb amputations has evolved at a much slower pace. Traditionally amputation surgery was viewed as a limb salvaging procedure focused on adequate soft tissue coverage and limb preservation strategies (Markatos et al., 2019). This perspective transformed into a reconstructive approach with the production of bionic limbs and the possibility of high-fidelity control capabilities (Clites et al., 2019). A recent review by Herr et al. describes this new approach with the intention of advocating for optimization of the residual limb and reconstructive techniques (Herr et al., 2020). Focus has shifted from preservation of maximal limb length to preservation of adequate limb length for prosthetic control (Sanders and Fatone, 2011). It is now recognized that maximal limb length does not necessarily equate to maximal function and in some instances, a shorter residual limb length may be preferable for superior prosthetic design. The reconstructive approach prioritizes the care of peripheral nerves, soft tissue, and residual muscles in an effort to improve pain control and provide a foundation for neural communication in a prosthetic limb (Brown et al., 2014). The design and implementation of this technology is dependent on the execution of the amputation procedure and subsequent limb reconstruction.

While considerable progress has been made in improving prosthetic technology and motor control, incorporation of sensory feedback is lacking. Sensation is responsible for enhancing motor control and restoring a sense of embodiment that contributes to overall function and quality of life in amputees (Witteveen et al., 2012; Tan et al., 2015; Tyler, 2015; Dornfeld et al., 2016). Without sensation, individuals are unable to recognize the position of their limbs in space and thus must rely on visual cues as their only source of feedback. Prior to the development of neural interfaces, non-invasive substitution methods such as electro-tactile or vibro-tactile feedback were created as a means of restoring sensation. Though these techniques have demonstrated some capability of sensory stimulation, they are not able to offer the level of selectivity necessary for clinical application. As a result, more invasive methods with implantable nerve electrodes and neural interfaces have been pursued (Ghafoor et al., 2017). The direct neural contact allows for more selective and specific stimulation; however, this stimulation comes at the expense of stability and overall durability (Navarro et al., 2005; Wurth et al., 2017). Long term implementation of these devices is dependent on establishing a balance between the conflicting forces of specificity and stability. This requires a multidisciplinary approach that integrates the fields of biomedical engineering, surgery, and prosthetic device design to address the challenges posed by these interfaces and propose more innovative ways to interact with the peripheral nervous system.

The ability for human and machine to communicate directly *via* the nervous system became a reality when peripheral neural interfaces (PNI) emerged. The interface design uses an individual's own peripheral nerve as a channel for signal relay between the brain and an external device. Each peripheral nerve is made up of both afferent and efferent fibers that allow for bidirectional communication. The afferent fibers transmit information *to* the nervous system and the efferent fibers transmit information *away* from the nervous system (Tyler et al., 2015). An electrode(s) is used to harness the electrical energy produced *via* these pathways for the stimulation or recording of signals required for bidirectional communication and control of a prosthesis.

Electrical signals generated by the peripheral nervous system can be used to power a prosthesis both directly and indirectly. Myoelectric systems record Electromyography (EMG) signals indirectly through surface electrodes to activate motor commands, whereas more invasive PNIs use direct contact with the nervous system to record these signals (Kung et al., 2013). Though these systems have significantly improved prosthetic control, they are far more limited in their ability to incorporate sensory feedback, a key component of bidirectional signaling required for closed loop control (Micera and Navarro, 2009; Carey et al., 2015).

The substantial progress that has been made in the field of neuroprosthetics and the formation of silos is reflected in the robust body of literature published on these topics. Many of the reviews focus on a specific area of the field such as electrodes (Rijnbeek et al., 2018; Russell et al., 2019; Raspopovic et al., 2020); interface design (Larson and Meng, 2020), or surgical techniques like Targeted Muscle Reinnervation (TMR) (Mioton and Dumanian, 2018; Oh and Carlsen, 2019; Peters et al., 2020); and Regenerative Peripheral Nerve Interface (RPNI) (Santosa et al., 2020). Others explore algorithmic control systems (Wolf et al., 2020); prosthetic device designs (Naufel et al., 2020); and specific outcomes such as sensation or myoelectric control (Tyler, 2015; Geethanjali, 2016; Ghafoor et al., 2017; Sensinger and Dosen, 2020; Raspopovic et al., 2021). The former comprehensive reviews are outdated and do not cover newer developments such as the Agonist-antagonist Myoneural Interface (AMI) or Osseointegration (OI) (Navarro et al., 2005; Kung et al., 2013; Bates et al., 2020; Vu et al., 2020a; Yildiz et al., 2020). Additionally, we see each of these reviews as important focal points highlighting gaps and advances within individual domains, without necessarily considering how these domains integrate with each other to form a cohesive solution or standard of practice.

In this review, we will discuss a global overview of the current state of peripheral nerve interfaces including nerve electrodes, surgical innovations, and available prosthetic technology. We explore the approach to amputations and the barriers that prevent clinical use of advanced prosthetics. The main goal of this review is to identify the strengths and weaknesses of various peripheral nerve interfaces in an effort to advocate a more collaborative approach that supports a combination of different electrical and surgical interventions to optimize utilization of the bionic limb.

## Electrodes

Electrodes interact with the peripheral nervous system myoelectrically or through nerve electrodes with varying degrees of invasiveness (Woo et al., 2016). This section of the review will focus on the electrodes that have direct contact with nerves. The reliability and longevity of a peripheral nerve interface depends on a number of electrode characteristics and requirements that contribute to the overall design. This includes tissue interaction, stimulation and recording capability, host immune response, and biocompatibility (Raspopovic et al., 2020). The area of greatest concern is electrode-tissue interaction leading to mechanical mismatch and a biological tissue response that results in device failure. Over time, micromotion within the tissue can lead to degradation of signal and potential neuronal damage (Woeppel et al., 2017). Similarly, the implanted foreign materials may trigger an inflammatory reaction that also contributes to such damage (Grill et al., 2009). Strategies to mitigate these challenges include reducing size of implant, constructing soft and flexible electrodes, and use of bioactive or conducting polymer coatings (Diment et al., 2018; Wellman et al., 2018).

The main goal of creating a successful, implantable electrode is establishing high quality signal transmission that is able to endure a surrounding harsh environment without significant tissue disruption. Ideally, this would allow electrodes to record motor potentials and evoke sensory signals with high selectivity and longevity. Selectivity is defined as “the ability to activate one population of neurons without concomitant activation of another” (Grill et al., 2009). This precise activation of different fascicles leads to the production of refined motor movements and natural discrimination of sensory precepts. Longevity refers to the stability—or activation of the same population of axons over time—and is critical for survival of the electrode and production of naturalistic sensation (Ghafoor et al., 2017). Selectivity and stability largely depend on invasiveness of electrodes and unfortunately act in opposition to each other. Selectivity tends to increase with invasiveness whereas stability tends to decrease. In addition, selectivity and stability are influenced by the number and configuration of electrodes (Lee et al., 2017; Charkhkar et al., 2018); biocompatibility (Woeppel et al., 2017); and pattern recognition algorithms (Hargrove et al., 2017). These properties significantly impact the stimulating and recording capabilities of electrodes and their ability to restore function (Merrill et al., 2005; Cogan, 2008). The fundamental electrochemical properties that dictate the actions of stimulating and recording electrodes are beyond the scope of this review.

The peripheral nerve electrodes can be separated into three different categories: extraneural electrodes, intraneural electrodes, and regenerative electrodes.

### Extraneural Electrodes

Extraneural electrodes, sometimes referred to as “epineural” electrodes, encircle the outside of the nerve and do not penetrate the epineurium. They are the least invasive nerve electrodes and are therefore less susceptible to damage (Micera et al., 2008). The cuff electrode has an insulated sheath with electrode contacts on the surface of the epineurium for recording and stimulation of large neural fibers (Loeb and Peck, 1996). There are several derivations of the cuff electrode that have been designed to improve selectivity and spatial resolution. These variations involve refining geometry (Dweiri et al., 2016); increasing electrode contact density (Polasek et al., 2009); and reducing nerve compression (Lee et al., 2017) to avoid subsequent damage. The flat interface nerve electrode (FINE) increases surface area, bringing axons to the surface and creating additional contact sites for selective stimulation (Tyler and Durand, 2002). Though this was an improvement from the traditional cuff electrode design, the FINE still requires high stimulation currents which may evoke unnatural feelings or paresthesias (Leventhal and Durand, 2003). The composite flat interface nerve electrode (c-FINE) design further reshaped the cuff electrode with areas of alternating flexibility and stiffness, creating an adaptable electrode with high contact density sites (Freeberg et al., 2017).

Extraneural electrodes have demonstrated chronic stability with recording and stimulating in humans for up to 11 years (Fisher et al., 2009; Tan et al., 2014; Christie et al., 2017; Charkhkar et al., 2018). Selectivity is expected to be limited due to lack of invasiveness and activation of larger afferent populations, creating cross talk from EMG activity of surrounding muscles and undifferentiated neural activity from neighboring fascicles. The recorded electroneurography (ENG) signals have lower amplitudes with poor signal to noise ratio (SNR) compared with EMG signals (Micera et al., 2010). Different strategies using machine learning algorithms and spatial filtering for separation of individual signals have been devised to reduce noise and optimize recording with extraneural electrodes (Wodlinger and Durand, 2011; Tang and Durand, 2014; Dweiri et al., 2016; Aristovich et al., 2018). Additionally, electrode configurations (Howell et al., 2015) and varied stimulation parameters (Cogan et al., 2016) have been designed to improve stimulation specificity. Tan et al. demonstrated that sophisticated patterns of stimulation can produce highly localized sensory precepts of several different qualities. These electrodes were the first to achieve restoration of meaningful sensation with long term stability and have the greatest potential for highly selective stimulation (Tan et al., 2014). Although these methods offer improvements in electrical communication, extraneural electrodes have not been able to provide complex control.

In addition to controlling movement and eliciting sensation, extraneural electrodes have also been investigated for use in pain management in amputees. Stimulation with the cuff electrode eliminated chronic phantom pain within 6 months with pain relief continuing even in the absence of stimulation. Similarly, the FINE electrode demonstrated elimination of phantom limb pain (PLP) after 9 months (Tan et al., 2015). More quantitative and comparative studies are needed to draw conclusions regarding the effectiveness of electrodes and reduction in PLP.

### Intraneural Electrodes

Intraneural electrodes penetrate the epineurium and directly communicate with nerve fascicles. There are three main intraneural electrodes: the longitudinal intra-fascicular electrode (LIFE), the transverse intra-fascicular multichannel electrode (TIME), and the Utah Slanted Electrode Array (USEA) (Jung et al., 2018). The original LIFE design places a stiff wire longitudinally along a set of fascicles; one wire is required for each electrode (Zheng et al., 2003). Similar to the early cuff electrodes, the LIFE's limited geometric flexibility and increased risk of damage led to the development of more advanced versions (Lawrence et al., 2004; Lago et al., 2007). The upgraded thin-film LIFE (tf-LIFE) uses flexible polyimide material and a higher density of electrode contacts compared to the original design. The additional sites increase activation of a specific set of fascicles; however, the longitudinal arrangement prevents access to different populations of fascicles (Kundu et al., 2014) and thus may limit selectivity compared with other intraneural electrodes. The TIME was instead purposefully designed in a transverse orientation and therefore can provide multiple contacts for different sets of axons at the same time (Boretius et al., 2010). The most invasive intraneural electrode, the USEA, is a penetrating microelectrode array containing 100 contact sites that is implanted transversely through the nerve to enhance selectivity and spatial resolution (Davis et al., 2016). The vast number of contact sites within different populations of fascicles offers the possibility of precise control of fine motor movements.

While the intraneural electrodes' intimate contact permits high selectivity with stimulation and increased signal amplitude with recording, it puts the nerve at risk for damage (Vu et al., 2020b). The main limiting factor of these electrodes is stability. Over time, the SNR decreases, and higher levels of stimulation are required (Raspopovic et al., 2020). LIFEs have had short term success with providing sensory feedback (Horch et al., 2011) and decoding grasp signals (Micera et al., 2011). A tf-LIFE implanted in a human amputee recorded stable motor signals throughout a 4-week trial, but after 10 days, sensory stimulation had diminished (Rossini et al., 2010). More recently, this stability has increased to 11 weeks with closed loop control of slippage and grasping (Zollo et al., 2019). The TIME has shown more promise, with numerous studies demonstrating its ability to provide selective stimulation and sensory feedback (Raspopovic et al., 2014; Oddo et al., 2016; D'Anna et al., 2019). The TIME's longevity is superior to the LIFE with demonstration of stability of stimulation signals for 6 months in three transradial amputees (Petrini et al., 2019b). Futhermore, the TIME has also been used in lower limb amputees, with findings showing increased mobility and improved confidence when sensory feedback is supplied (Valle et al., 2021). This type of closed loop control is especially important in lower limb amputees who lack balance and native gait cues.

Among the intraneural electrodes, the USEA has the greatest potential for achieving high selectivity. Two transradial amputees with implanted USEAs were able to control individual finger movements as well as elicit multimodality sensory percepts (Davis et al., 2016). USEA implanted in two other transradial amputees achieved independent control of five degrees of freedom (DoF) and perceived as many as 131 sensory percepts. EMG signals in addition to the ENG signals were required to achieve this level of control (Wendelken et al., 2017). Though signals tend to degrade over time, performance of USEAs continue to improve with recent evidence of stability and functionality after 14 months (George et al., 2020). These electrodes are capable of recording and stimulating individual neural fascicles and thus have the potential to provide complex control of a prosthesis. They must demonstrate long-term stability of signals before they will be accepted for chronic implementation in humans.

Similar to the extraneural electrodes, the intraneural electrodes have demonstrated success in mitigating postamputation pain. Using LIFEs for sensory feedback resulted in resolution of symptoms due to phantom-limb syndrome (Rossini et al., 2010) and neural stimulation of TIME resulted in decreased phantom limb pain (Petrini et al., 2019a). This was also seen long term with USEA in a transradial amputation with demonstration of phantom pain reduction and prosthesis embodiment (Page et al., 2018). This was achieved with open loop motor control and open loop sensory control idependently.

### Regenerative Electrodes

Regenerative electrodes are considered the most invasive PNIs with the largest number of axon contacts. They are classically defined as requiring transection of the nerve so it can regenerate around or through an electrode (Lago et al., 2005). However, this definition is a byproduct of the experimental methodology, which is based on the standard neurorrhaphy model in an intact limb, measuring return of functions (Vela et al., 2020). In the amputation setting, the invasive transection is typically the result of the traumatic event, or an operative requirement of the amputation. Furthermore, treatment for post-amputation neuroma typically involves surgical dissection and excision of the neuroma (Souza et al., 2014; Woo et al., 2016; Israel et al., 2018) before the final surgical intervention to prevent its return. In the amputation setting, the application of regenerative electrodes to a transected nerve end adds little to no invasiveness to the amputation procedure itself (Millevolte et al., 2021). There are various design types including sieve electrodes, regenerative multi-electrode arrays, and scaffolding electrodes. These electrodes have the potential to provide the highest level of selectivity with low stimulation thresholds and further improve prosthetic control (Grill et al., 2009; Cutrone et al., 2015; Ghafoor et al., 2017; Coker et al., 2019). However, there are a number of challenges that make implementation of regenerative electrodes difficult. The transected nerve fibers must regenerate appropriately and successfully integrate with the electrodes. Regenerative electrodes have demonstrated evidence of stable recording and stimulation of signals in animals (MacEwan et al., 2016), though the concern for damage has prevented their use in humans.

## Surgical Techniques

Peripheral nerve interfaces offer promising potential for the future of advanced prosthetics; however, the majority remain experimental, facing obstacles they must first overcome to achieve full clinical translation. The surgical application of PNIs is one such obstacle, particularly in relation to the application of more invasive PNIs available. As a result, unique surgical techniques have been developed to address these limitations and enhance functionality of the interface. They are designed to create high fidelity signals capable of simultaneous control with multiple degrees of freedom (Kung et al., 2013). Increased opportunity for complex movements motivated the prosthetic industry to maximize range of motion (ROM) and revolutionize device attachment.

These procedures not only provide alternative methods of prosthetic control, but can also prevent and treat painful postamputation neuromas (Santosa et al., 2020). After transection, a peripheral nerve works to regenerate until it finds an end organ to innervate. In the setting of amputations, the nerve undergoes axonal sprouting and regeneration without available target sites, and thus there is formation of an unpredictable, excitable mass of fibers (Stokvis et al., 2010). Chronic neuroma pain is a debilitating sequela of amputation that often precludes amputees from using a prosthetic device and significantly contributes to loss of function. Some studies suggest that at least 25% of amputees experience painful neuromas (Sehirlioglu et al., 2009; Bowen et al., 2017). Prior to these innovative surgical techniques, over 100 different treatments were unsuccessful in establishing a standard solution for relief of symptoms, with at least 20-30% of cases being refractory to treatment (Poppler et al., 2018).

In addition, these surgical techniques have encouraged conversations about amputations and have helped shape the way we think about them in the traditional landscape (Herr et al., 2020). There is a fundamental need to redefine the amputation surgery that places an emphasis on reconstruction as well as on incorporation of neural interfaces for prostheses.

### Targeted Muscle Reinnervation (TMR)

**Figure 1:**
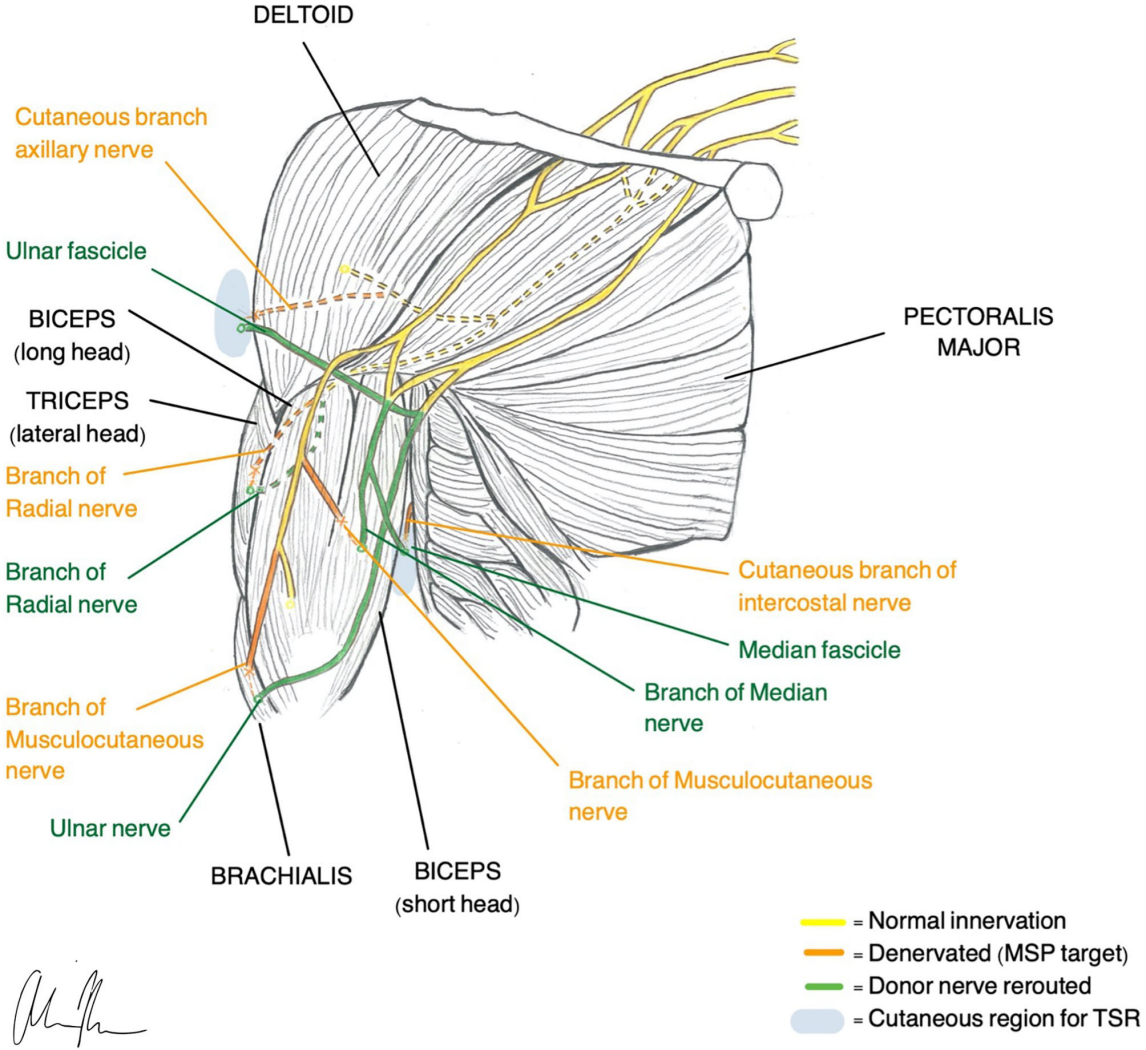
Illustration of TMR (Gart et al., 2015) and TSR (Hebert et al., 2014) construction in a transhumeral amputee.

TMR was one of the first surgical techniques established and was originally designed to be an improvement of the myoelectric systems already used for prosthetic control. These traditional methods typically only allow for control of one DoF at a time and do not provide any sensory feedback or treatment of neuropathic pain (Kilgore et al., 2008). TMR reroutes residual nerves from the amputated limb to different, denervated target muscles that are functionally not required or functionally redundant. The native nerve is transected near the donor muscle and then coapted to the donor nerve at the muscle entry point (Figure 1). The denervated target muscles lose their native function and are instead transformed into biological amplifiers that allow for simultaneous motor control of multiple DoF (Kuiken et al., 2004, 2009). TMR can even provide prosthetic movements from muscles that are no longer present.

#### Motor Control

Unlike the other myoelectric systems, TMR provides more natural control without unnatural code switching or requirement of intact distal muscles for activation of movement (Kuiken et al., 2007). Attempted movements produce EMG signals in the reinnervated muscles that are measured by surface electrodes and used to power a prosthesis. Although effective in recording EMG activity, there are several problems associated with surface electrodes. EMG signals from nearby muscles create noisy cross talk and subsequent difficulty with signal extraction. In addition, there is limited available surface area and any skin disturbances may cause the electrodes to shift (Young et al., 2011; Kuiken et al., 2016). Despite these challenges, surface electrodes provide a method of EMG signaling that is non-invasive and free of percutaneous wiring.

Transhumeral amputees and individuals with shoulder disarticulations are the most common recipients of TMR, although recently there has been more work looking at transradial amputees (Pierrie et al., 2018). In general, TMR in the upper extremity offers four different control sites that are each reinnervated by one of the major peripheral nerves. For example, in transhumeral amputees, native innervation of the long head of the biceps and triceps remains intact while the short head of the biceps is reinnervated by the median nerve and the lateral head of the triceps is reinnervated by the distal radial nerve. Native innervation controls elbow flexion/extension and the new reinnervations control hand open/closure (Cheesborough et al., 2015). If enough residual limb is available, it is possible to re-route part of the ulnar nerve to the brachialis so wrist control can be achieved.

Patterns for reinnervation are determined by the availability of musculature and donor nerves (Gart et al., 2015). The number of control sites is limited by the number of available, denervated target muscles. Since nerve transfer involves reimplantation of a whole nerve only 4–5 functions are possible and thus complex control with intrinsic hand muscles is difficult to achieve (Smith et al., 2015). To obtain direct control with a naturalistic, intuitive feel, one EMG control must correlate with one DoF. Decreased spatial resolution and subsequent EMG cross talk makes this challenging (Farina et al., 2014). In an effort to increase the number of control possibilities and execute functions, pattern recognition algorithms have been applied to enhance signal interpretation (Zhou et al., 2007; Kuiken et al., 2009, 2016). A recent at-home trial with TMR patients demonstrated superior performance with pattern recognition compared with direct control (Hargrove et al., 2017). Although pattern recognition addresses some of the shortcomings of surface EMG, it does not provide the same natural feel as does direct control. Instead, movements must be carried out sequentially in specific patterns, and the lack of simultaneous and proportional control feels slow and unusual (Farina et al., 2014).

#### Sensory Feedback

To further improve motor control and close the loop in a bidirectional prosthesis, sensation must be restored (Tyler, 2015; Markovic et al., 2018; Petrini et al., 2019a). An analogous sensory version of TMR, called Targeted Sensory Reinnervation (TSR), has been presented as a potential solution to restoring sensation. Similar to TMR, this method transfers transected sensory nerves to the motor entry points so it can then grow into denervated residual skin (Kuiken et al., 2007). This allows an amputee to experience referred sensation from the amputated limb when the corresponding reinnervated area of skin is activated. TSR has demonstrated its ability to reinnervate skin and produce tactile feedback in several studies (Kuiken et al., 2007; Kim and Colgate, 2012; Hebert et al., 2014). However, results with TSR have been variable, and several limitations have prevented full adoption of the method (Hebert et al., 2016). The reinnervated skin overlies muscles that produce additional EMG signals, creating noise and making independent signal extraction challenging. The need for both touch feedback tactors and EMG electrodes requires a large amount of skin surface area (Schofield et al., 2020). Additionally, motor efferent signals predominate over afferent and thus simultaneous control of movement and sensation is not possible (Kim and Colgate, 2012). When tactile feedback is produced, it may feel unnatural since the individual must acknowledge that this sensation is coming from a different part of the body. Studies of upper limb cortical maps in individuals with TSR indicate that integration of sensory information does not occur with visual bodily clues (Serino et al., 2017). Despite the ability of TSR to elicit sensory precepts, cortex integration requires an interpretation of sensation that is otherwise unnatural.

#### Management of Symptomatic Neuromas

Although TMR has not demonstrated significant success with restoring sensation, it is one of the most effective methods of managing symptomatic neuromas in amputees. The denervated target muscle provides a destination for a regenerating nerve to naturally grow into without formation of aberrant axons and subsequent pain (Mioton and Dumanian, 2018). These findings have translated clinically and patients with neuroma pain who underwent TMR experienced resolution or reduction in their pain postoperatively (Souza et al., 2014; Bowen et al., 2019). TMR is not limited to secondary intervention and can also be performed preemptively at the time of amputation. Studies assessing TMR as a primary intervention have demonstrated reduced phantom limb pain, improved outcomes related to pain quality, and lower rates of opioid use (Valerio et al., 2020; O'Brien et al., 2021). Similarly, a study of primary TMR in below knee amputations (BKA) did not find that any patients developed symptomatic neuromas during the follow up period. Additionally, early phantom limb pain was significantly reduced by 3 months and resolution was seen by 6 months (Bowen et al., 2019). Further investigation with a multi-institutional randomized clinical trial revealed that primary TMR improved phantom limb pain and trended toward reduced residual limb pain compared to standard neurectomy (Dumanian et al., 2019).

#### Surgical and Prosthetic Applications

Neuroma treatment and prevention with TMR has been used in both upper and lower extremity amputations. In terms of prosthetic control, TMR has only been used in individuals with upper extremity amputations (Oh and Carlsen, 2019; Peters et al., 2020). However, anatomic studies have identified transfer patterns and potential motor targets in BKAs (Fracol et al., 2018) and transfemoral amputations (TFA) (Agnew et al., 2012). For either application TMR can be performed as a primary surgery at the time of amputation or as a secondary surgery post amputation.

Unlike many PNI surgical techniques, TMR utilizes non-invasive electrical connections *via* surface electrodes, permitting take-home use and participation in clinical studies (Schofield et al., 2020). With new technology it may soon possible to replace these surface electrodes with implantable myoelectric sensors for enhancement of motor control and improvement of sensory feedback (Lowery et al., 2006; Miller et al., 2008). A recent prospective study of three transhumeral amputees with chronically implanted myoelectric sensors (IMES) demonstrated substantially improved functional outcomes compared with standard surface electrodes (Salminger et al., 2019). This provides high quality signals and improved performance in direct control without the need for pattern recognition. IMES represents a significant achievement for PNIs using intramuscular electrodes, thought it does not come without limitations. The current design is not compatible with osseointegration or shoulder disarticulations and is restricted to 6 total sensors, or a maximum of 3 DoFs (Salminger et al., 2019). TMR may be used in conjunction with other invasive nerve electrodes or even osseointegration for implementation of a fully implantable system.

### Regenerative Peripheral Neural Interface (RPNI)

**Figure 2:**
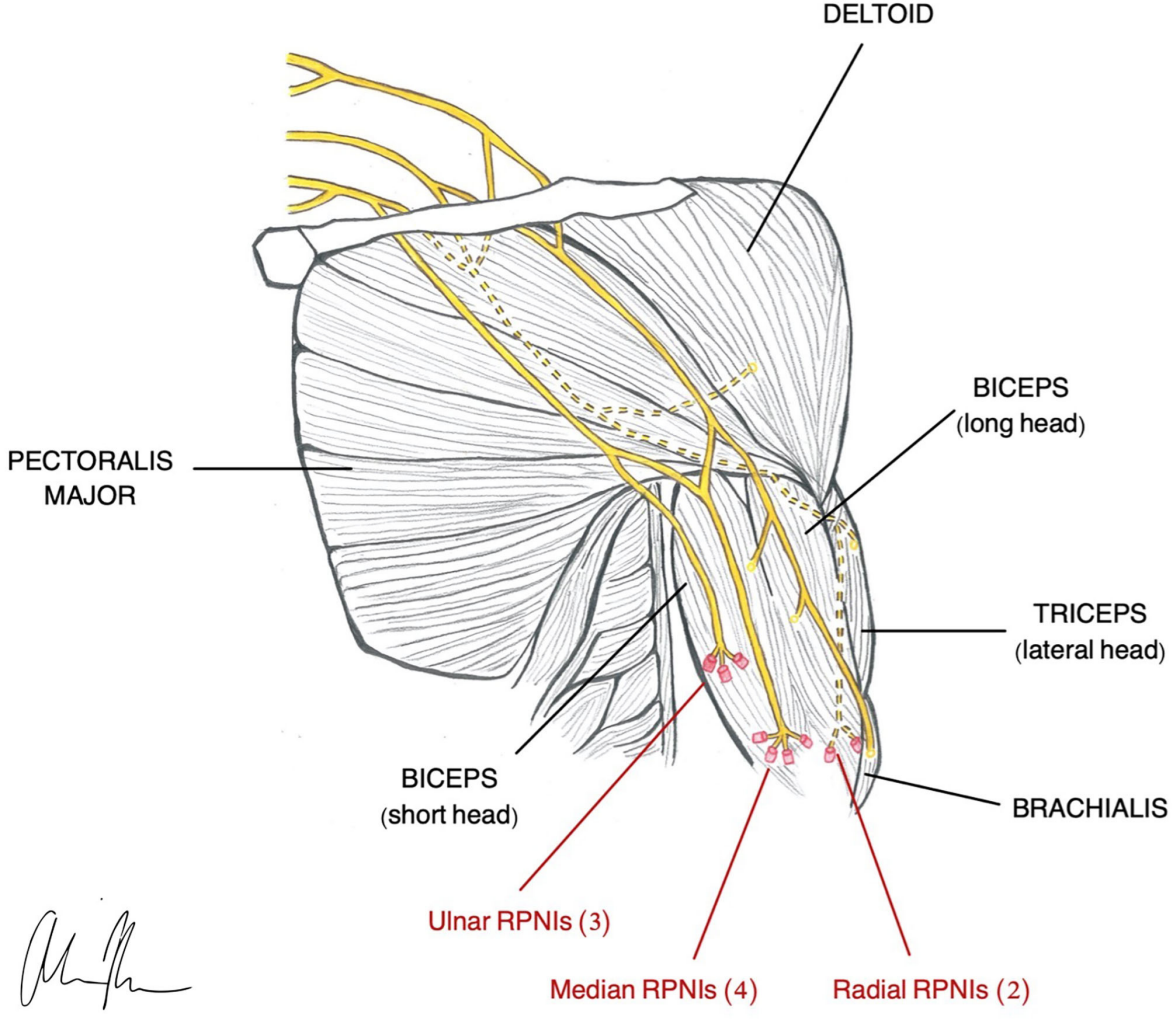
Illustration of RPNI construction in a transhumeral amputee (Vu et al., 2020a).

RPNI is based on the same neurobiological foundations as TMR in that it uses a transected peripheral nerve or fascicle and implants it into an autologous muscle graft (Figure 2) to serve as a biological amplifier of efferent signals (Santosa et al., 2020). The novel aspects of the RPNI are that it utilizes free muscle grafts that contain integrated electrodes to improve specificity and reduce crosstalk, thereby producing high quality, isolated EMG signals (Urbanchek et al., 2012, 2016; Kung et al., 2014; Woo et al., 2014; Irwin et al., 2016; Ursu et al., 2016). Furthermore, RPNI does not require denervation of existing muscles and is therefore not restricted to a limited number of control sites. Its ability to interface with multiple fascicles gives RPNI the potential to control for many DoFs. RPNI, like TMR, was initially developed as a means of advancing prosthetic function and was later discovered to serve as a viable treatment for symptomatic neuromas. Substantial preclinical evidence demonstrating RPNI's ability to produce stable efferent signals has resulted in clinical translation of the RPNI to treat amputation neuroma and prosthetic control in humans.

#### Motor Control

The RPNI was created with the purpose of expanding myoelectric interface capabilities and achieving greater signal specificity with chronic stability. The RPNI is designed to provide complex intuitive movement by maximizing number of control sites and increasing DoF. In a pilot study by Vu et al., RPNIs in upper limb amputees generated large amplitude EMG signals with high SNR and chronic stability over 10 months. This allowed for real-time continuous and simultaneous control of one DoF finger and two DoF thumb movements, as well as control of missing intrinsic hand muscles (Vu et al., 2020b), making RPNI the first PNI in which control of individual finger movements could be demonstrated.

This type of selectivity is possible with RPNI because of the way it utilizes free muscle grafts and is not limited by availability of residual native muscles. In addition, implantable muscular electrodes provide specific EMG signals with minimal cross talk, enhancing selectivity (Farina et al., 2014). To further maximize prosthetic function, electrodes may be implanted in residual muscles for additional control sites and improvement of signal specificity (Vu et al., 2020b). Similarly, application of control algorithms used in other PNI designs may help enhance interpretation of fine movement.

#### Sensory Feedback

There is currently no published data available regarding RPNIs and restoration of sensation.

#### Management of Symptomatic Neuromas

In addition to providing prosthetic control, RPNI is a novel surgical technique capable of preventing and treating symptomatic neuromas. Similar to TMR, it provides denervated target muscles for regenerating axons to grow into, thereby reducing the number of available axons for neuroma formation (Kung et al., 2014).

The first human study of RPNI for neuroma relief was a retrospective case series of 16 amputees. Findings revealed a 71% and 53% reduction in neuroma pain and phantom pain, respectively (Woo et al., 2016). Patients also reported decreased pain interference, reduction in opioid use, and improved prosthetic use (Woo et al., 2016). A more recent retrospective study investigated RPNI as a prophylactic intervention for the prevention of neuromas. The study compared postamputation pain outcomes between individuals who underwent prophylactic RPNI at the time of amputation to individuals who underwent amputation without RPNI and thus served as controls. The patients with RPNI showed 0% incidence of symptomatic neuroma formation (13% in control group) as well as a significant reduction in phantom limb pain compared with the control group (Kubiak et al., 2018). The results of these initial studies suggest that RPNI is efficacious in both the prevention and treatment of symptomatic neuromas and phantom pain.

#### Surgical Application and Prosthetic Compatibility

RPNIs can be implemented at the time of amputation or at a later date with a second operation. Although it may be more beneficial to do prophylactically for neuroma prevention, electrode implantation may dictate the timing of the surgery. A recent study by Srinivasan et al. hypothesized that electrical stimulation would expedite the process of revascularization and regeneration in free muscle grafts; however, the group instead discovered that stimulation actually interferes with these processes (Srinivasan et al., 2019). Future work will be needed to determine optimal timing for surgery based on these results.

RPNI can be used in upper and lower extremity amputations for symptomatic neuroma management (Woo et al., 2016; Kubiak et al., 2019), but in terms of prosthetic control, current studies are limited to upper extremity amputations. The devascularized free muscle grafts used for each RPNI do not require denervation of any native muscles nor depend on long nerve transfers (Woo et al., 2014), and thus could theoretically be applied to any amputation level in any limb. Previous studies have used the vastus lateralis as the autologous donor muscle (Vu et al., 2020b), though future work may explore other options. Smaller grafts have demonstrated better signal production and tissue viability compared with larger grafts in rat models (Hu et al., 2021). Current research is underway to determine optimal size, location, and number of RPNIs that can be used.

Relative to other PNI designs, surgical implantation of RPNI is relatively straightforward. It does not require microsurgery techniques and is likely translatable across multiple surgical subspecialties (Kubiak et al., 2018). This should help expedite the adoption of RPNI as an established neuroma treatment. By contrast, there are barriers that preclude current clinical use of RPNIs for prosthetic control. For instance, the use of implantable electrodes requires percutaneous wiring that presents a risk for infection, breakage, and disrupted connection (Ortiz-Catalan et al., 2014). For take-home use, it will be essential that RPNIs operate *via* surface electrodes, wireless IMES, or in combination with osseointegration. Lastly, it will be necessary for RPNIs to incorporate sensory feedback for complete bidirectional control. The current design does not have a specified method of providing this feedback and therefore will likely require use in conjunction with other PNIs to restore sensation.

### Agonist-Antagonist Myoneural Interface (AMI)

**Figure 3:**
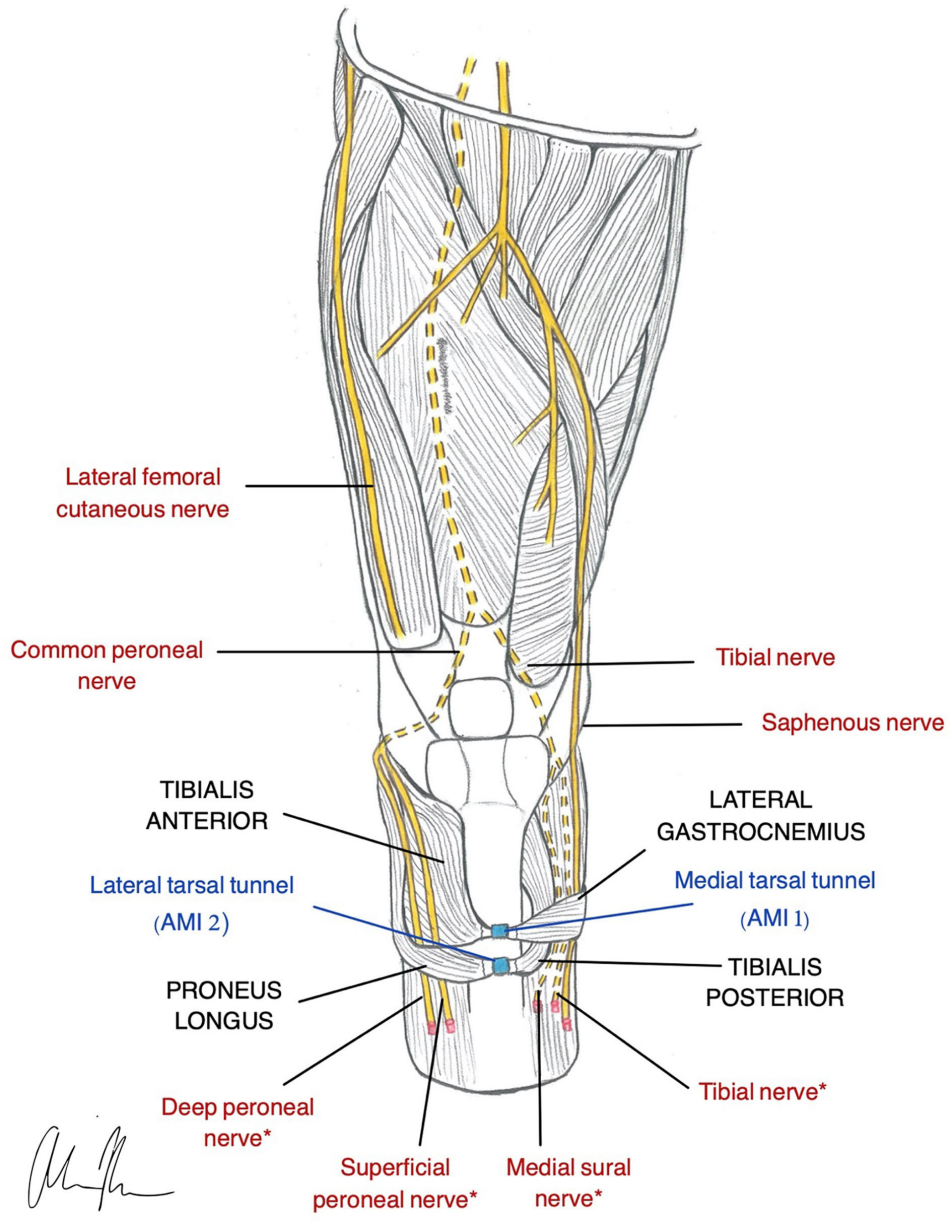
Illustration of AMI construction in a transtibial amputee (Herr et al., 2020). Native relationship between the Lateral Gastrocnemius and Tibialis Anterior is restored using the medial tarsal tunnel (AMI 1). The Tibialis Posterior and Peroneus Longus native relationship is restored using the lateral tarsal tunnel (AMI 2). *nerves are used for RPNI for motor control.

The AMI is the newest PNI surgical innovation represented in the literature. It is an amputation model designed to incorporate native residual muscles and corresponding neural signals for supplementation of proprioceptive feedback and joint control (Clites et al., 2017). Agonist-antagonist muscle pairs in the residual limb are surgically coapted in series so that agonist contraction results in antagonist stretch (Figure 3). Activation of mechanoreceptors in these muscles generates proprioceptive signals that provide the CNS with information related to joint movement. One AMI construct correlates with one DoF and thus control of a prosthesis with two joints requires two AMIs (Clites et al., 2018). Preservation and utilization of the native muscle relationships makes the AMI a unique and promising design for bidirectional communication in a prosthesis.

There are a number of different ways an AMI and its corresponding prosthetic joint can be controlled. This is largely dependent on the viability of residual limb. Herr et al. outlines the recommended surgical approaches for BKAs based on availability of distal tissues. If these tissues are fully intact, the AMIs can be controlled through the native neural pathways provided by the agonist-antagonists. If tissue availability is limited, it may be necessary to use alternative approaches such as TMR or RPNI in combination with the AMI for control (Herr et al., 2020). Lastly, if there is no residual tissue remaining, TMR, RPNIs, or 2-stage AMI is used (Srinivasan et al., 2019). If accessible, the AMI muscle pairs are mechanically linked using the tarsal tunnels from the amputated limb. Otherwise, they may be created artificially. Additionally, in any of these scenarios, neurovascular island flaps may be created to improve sensation and fit within a socket prosthetic (Herr et al., 2020). These complex surgical techniques incorporate many of the PNI designs already discussed in this review.

#### Motor Control

The AMI utilizes native spindle fibers and Golgi tendon organs in paired muscles to restore both motor control and proprioceptive feedback. Although studies are limited, early data suggests that the AMI is capable of generating high quality efferent signals, integrating reflex arcs, and preventing disuse atrophy (Clites et al., 2018; Srinivasan et al., 2019). When compared with four traditional amputees, an AMI patient exhibited natural reflexive behaviors and improved prosthetic control (Clites et al., 2019). This suggests that proprioceptive feedback not only restores sensation, but also enhances joint control and prosthetic function.

#### Sensory Feedback

The most promising application of the AMI is the restoration of proprioception. While other interfaces such as the cuff electrode have provided some sensory capabilities, they have not successfully incorporated natural proprioceptive feedback (Tan et al., 2015). This is largely due to the complex interplay between mechanoreceptors in the skin, muscles, and joints that make proprioception difficult to achieve (Weber et al., 2012). Proprioception is a unique sensation of which humans are not consciously aware. It allows us to know where our body is in time and space without visual input. Though its mechanisms are not well-understood, the significance of proprioception in motor control and joint stability is well-established (Proske and Gandevia, 2012). This capacity allows us to adapt to changes in the external environment and assists us in the planning of motor commands (Riemann and Lephart, 2002). Furthermore, it contributes to the sense of embodiment necessary for our limbs to feel like they are a part of “us.”

The AMI takes advantage of the body's natural proprioceptive pathways by coupling agonist and antagonist muscle pairs to control and translate feedback from a prosthetic joint. This was first demonstrated in animal models with evidence of graded afferent signals in multiple studies (Clites et al., 2017, 2018; Srinivasan et al., 2019). In humans, the AMI has demonstrated closed-loop joint torque control (Clites et al., 2018), and functional neuroimaging has revealed proprioceptive activity similar to that of individuals without an amputation (Srinivasan et al., 2020). Though the AMI does not provoke natural sensory precepts such as touch, the ability to restore proprioception is undoubtedly just as important a capability.

#### Management of Symptomatic Neuromas

There is currently minimal published literature discussing the AMI in terms of symptomatic neuromas and postamputation pain. Unlike TMR and RPNI, the AMI was not designed based on amputation neuroma. In the first pilot study (Clites et al., 2018) none of the three patients reported phantom sensations or cutaneous pain post-operatively. However, the authors attribute this to the RPNIs that were created for transected nerves. Although the AMI may not directly prevent or treat symptomatic neuromas, it is conceivable that preservation of native neuromuscular relationships may contribute to a reduction in phantom pain (Karl et al., 2001; Grüsser et al., 2004; MacIver et al., 2008). Future studies should further explore the role of AMI in the management of postamputation pain.

#### Surgical Application and Prosthetic Compatibility

Of all the proposed surgical techniques, AMI is the most complex and technically challenging approach. It requires an extensive operation (or several), longer hospital stay and recovery time, and a surgeon capable of performing such an advanced procedure (Herr et al., 2020). Thus, far, the AMI has only been used in humans requiring a primary BKA. Recently, Srinivasan et al. explored the possibility of implementing AMI as a secondary, revision surgery in patients who already underwent amputation. This dual staged operation is even more technically difficult than the original AMI approach. Regenerative AMIs are created by identifying and securing nerve fascicles in muscle grafts during the first operation. During the second stage, the appropriate flexor and extensor grafts are then coupled together for functional use (Srinivasan et al., 2019). This revision model provides evidence of viability, graded efferent and afferent signaling, and mechanical stability that is comparable to the original single stage approach (Clites et al., 2017). Surgical models for using AMI in AKAs have been created in animals and have the potential to be translatable in humans (Clites et al., 2019).

In terms of using AMI in Upper extremity amputations, no data is currently published. Given the complexity of upper extremity anatomy and fine motor control, it is likely that the AMI would need to be used in conjunction with TMR and/or RPNI for control of multiple DoF.

The AMI procedure offers an “alternative form of limb reconstruction” designed to augment the residual limb in preparation for a prosthesis. Current studies have used temporary fine wire electrodes for electrical connectivity (Herr et al., 2020). These are not applicable outside the lab and thus surface electrodes or implantable muscle electrodes (without external wiring) would be necessary for clinical use. Nonetheless, the AMI is the first technique designed to mimic the natural gait cycle which relies heavily on proprioception and coordination of fine muscle movements.

### Osseointegration (OI)/Osseointegrated Neural Interface (ONI)

Osseointegration is the direct skeletal anchorage of a metal implant to bone. Developed as a method of improving mechanical stability between an bone/implant interface, OI initially found success in the field of dentistry with tooth implants (Jacobs et al., 2000; Brånemark et al., 2001). Its application was eventually extended to extremity prosthetics in response to the challenges and complications associated with traditional socket prostheses. Unlike the socket-based prostheses, OI provides reliable mechanical stability, physiological load bearing, and increased range of motion (Al Muderis et al., 2018; Brånemark et al., 2019; Ackerley et al., 2020; Hagberg et al., 2020). This facilitates ease of use and promotes continued prosthetic use.

**Figure 4:**
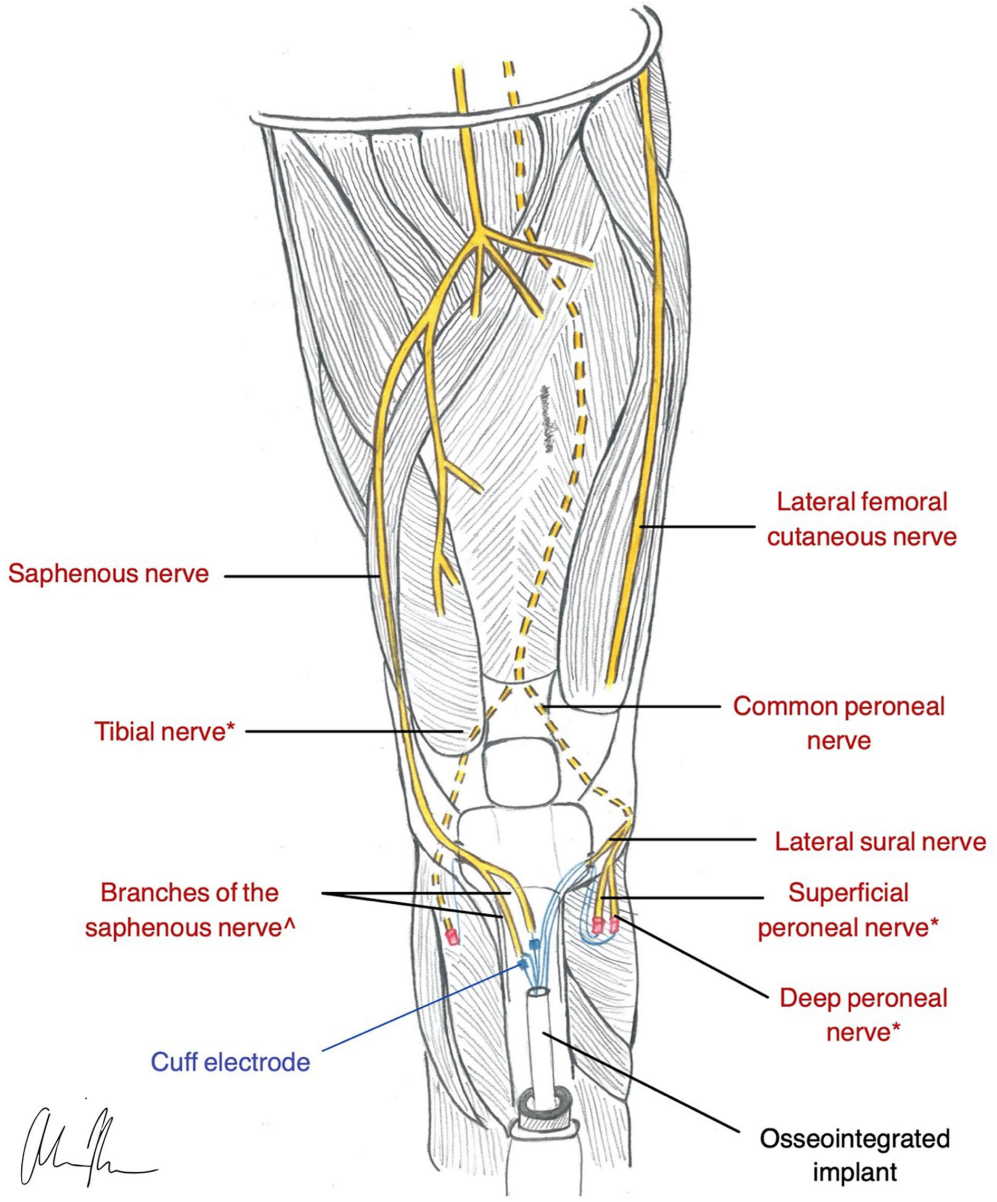
Illustration of ONI construction in a transtibial amputee (Dingle et al., 2020b). * nerves are used for RPNI for motor control. ^nerves are used with a cuff electrode for sensation and are inserted into medullary canal *via* corticotomy in the Tibia.

The principles of OI, combined with concepts of nerve regeneration, led to the creation of a novel peripheral nerve interface design, the Osseointegrated Neural interface (Figure 4). Dingle et al. built this interface based on the idea that the intramedullary canal can provide a stable and protective environment for nerve regeneration (Dingle et al., 2020b). Although transposing nerves into bone was established as a treatment for symptomatic neuromas almost eight decades ago (Boldrey, 1943), the ONI takes the application one step further, demonstrating the intramedullary environment's ability to provide the stability required for the implementation of more selective, invasive electrodes (Dingle et al., 2020b). Like TMR and RPNI, the ONI is rooted in the treatment of symptomatic neuromas, while its application for prosthetic control remains experimental.

#### Motor Control

The ONI has not yet been evaluated in humans, but animal studies have provided promising results. A rabbit model with an intramedullary cuff electrode demonstrated evidence of nerve regeneration with the ability to produce efferent and afferent signals over 3 months (Dingle et al., 2020a). If this result can be translated to even more invasive electrodes such as intraneural or regenerative electrodes, the ONI may be capable of providing bi-directional signaling with high fidelity control. Additionally, the increased range of motion provided by OI could further supplement functionality.

#### Sensory Feedback

Osseointegration is capable of producing sensation on its own through the mechanical stimulation generated from a bone anchored prosthesis. This has been termed osseoperception or osseoproprioception and is thought to be an important factor in prosthetic usability (Jacobs et al., 2000; Klineberg, 2005). The underlying mechanisms that contribute to osseoperception are not well-understood and remain a subject of debate (Mohan Bhatnagar et al., 2015). Despite this uncertainty, there is evidence in the literature demonstrating the benefits of osseoperception. Subjectively, osseoperception has been identified as an improved perception of the environment with an increased awareness of a prosthesis (Jacobs et al., 2000). When compared with individuals with socket prostheses, those with an osseointegrated prosthesis experience an increased sensitivity to vibration (Häggström et al., 2013; Clemente et al., 2017). Furthermore, numerous studies have shown that osseointegration is associated with improved patient reported outcomes, better prosthesis-associated quality of life, and increased mobility (Brånemark et al., 2019; Hagberg et al., 2020; Pospiech et al., 2020).

Although osseoperception improves vibratory sensation and prosthetic embodiment compared with traditional socket users, it does not provide sufficient sensory feedback for restoring grasp behavior and improving motor coordination (Mastinu et al., 2019). The single abutment point in OI implants limits the available stimulation points for sensory feedback. However, Mayer et al. demonstrated that supplementary tactile feedback can be transmitted simultaneously through a single feedback channel to achieve adequate performance in these tasks (Mayer et al., 2020). This addresses the challenges of conveying information through one channel by improving its efficiency. Additionally, hearing has been shown to play a role in the improved sensation experienced by those with osseointegration, compared with those with a socket prosthesis (Clemente et al., 2017). Combing tactile feedback with hearing creates an additive effect that further improves prosthetic performance.

In relation to the ONI, there is additional potential for restoring sensation through the nerve electrodes housed in the intramedullary canal. As mentioned above, the ONI rabbit model was capable of both efferent and afferent signaling, and thus sensation could potentially be restored in this manner (Dingle et al., 2020b). The more invasive the electrodes are, the greater potential there is for establishing highly selective stimulation.

#### Management of Symptomatic Neuromas

The original design of the ONI was created on the basis of using the intramedullary canal of long bones to prevent symptomatic neuromas. This method was first established many years ago (Boldrey, 1943) and is based on the premise that the medullary cavity provides an insulated environment that can prevent a regenerating nerve from erroneously innervating muscles and skin. This prevents neuropathic pain by protecting the exposed nerve from external stimuli (Israel et al., 2018). The ONI not only takes advantage of neuronal regeneration for electrical signaling purposes but also for the prevention of symptomatic neuromas. Pain is prevented by placing axons in an environment that restricts neuronal regeneration and innervation to electrode sites rather than problematic areas such as skin.

#### Surgical Application and Prosthetic Compatibility

Osseointegration can be performed as a single stage operation (Al Muderis et al., 2017) or through a dual-staged approach. There is currently no standardized protocol for either strategy. Instead, the various surgical techniques tend be institution-based or implant dependent. Overall, the concept of OI is designed to be applied to amputations of any level. Although more attention has been given to the lower extremity amputations, it has been successfully used in upper extremity amputations as well (Jönsson et al., 2011). OI has been used throughout Europe and Australia for the last few decades; however, it was only recently approved in the United States by the Food and Drug Administration (FDA) *via* a humanitarian use device exemption. There are currently five implant options being used worldwide (Zaid et al., 2019).

As outlined above, individuals with OI experience superior outcomes compared to those with socket prostheses. However, there are challenges and potential issues related to OI that should be considered. The biggest concern with an OI implant is infection. Fortunately, the majority of OI- related infections are superficial and are successfully treated with outpatient antibiotics (Overmann et al., 2020). The risk of osteomyelitis is reported to be around 10% (Jacobs et al., 2000; Tillander et al., 2017). Nevertheless, management of the soft tissue interface is critical to preventing a deep infection and subsequent implant failure. Strategies for infection prevention involve preserving tissue adherence and tissue perfusion to optimize survival (Souza et al., 2020). Additional mechanical complications that may contribute to implant failure include loosening or failure of fixation and peri-implant fracture. A prospective study of 51 patients with TFA revealed a 5-year cumulative fixture survival rate of 92% a revision-free survival rate of 45%. Eleven patients experienced mechanical complications, with 3 implants requiring removal secondary to loose fixation (Brånemark et al., 2019).

Overall, the ONI has the potential be a revisionist surgery capable of improving functionality and maximizing usability through integration of a peripheral nerve interface and an osseointegrated implant for intuitive prosthetic control and prevention of neuropathic pain all in one procedure.

## Control Methods

### Myoelectric Control Options

Creating a system that is capable of naturalistic and intuitive prosthetic control is dependent on more than just interface design. It requires control methods that employ algorithms to interpret extracted EMG signals for the prediction of intended postures and movements. These non-invasive strategies can provide sophisticated control when there is poor selectivity of signals due to cross-talk from surface electrodes or if there is a lack of stability with invasive electrodes.

#### Conventional Control Methods

The most simple and common control method utilized in myoelectric devices is conventional or direct control. This uses two EMG signals from one muscle pair to control one DoF. Control of more than one DoF requires a mode switch with co-contraction of the muscle pair. This switching is cumbersome and can be cognitively challenging with increasing complexity (Young et al., 2014). Furthermore, direct control typically uses surface EMG electrodes, limiting the overall efficacy due to interfering signals from EMG crosstalk. In order to achieve simultaneous control of more than one DoF there must be at least four control sites available with minimal EMG cross talk (Farina et al., 2014). This is not possible in many upper limb amputees unless they have additional control sites through TMR or RPNI. As a result, this type of control can feel unnatural and is often restricted to simple movements (Hargrove et al., 2017).

#### Pattern Recognition Algorithms

In an effort to improve direct control, pattern recognition algorithms have been developed. This method identifies a pattern of signals provided by multiple electrodes and interprets it as a pre-defined movement or posture. This is particularly important for restoring function in higher level amputees as it has the ability to increase DoF despite missing residual joints. Although this provides intuitive control, it does not allow for simultaneous control of multiple motions. Instead, they must occur in sequence, limiting the ability to restore natural feel (Li et al., 2010). Furthermore, pattern recognition requires intensive training sessions and is cognitively demanding. It. depends on repetition of same the movements and thus any changes in the EMG patterns may lead to worsening performance.

Despite these limitations, there has been success with pattern recognition in the virtual and off-line setting (Hargrove et al., 2018), but very limited data to support its use in the clinical setting. The translation of off-line performance to online performance with real time feedback is uncertain (Farina et al., 2014; Hargrove et al., 2017). The potential deterioration of performance in an at-home setting poses a dangerous risk to both the individual and the integrity of the prosthetic device. However, Hargrove et al. recently demonstrated improved performance with pattern recognition compared to direct control in transhumeral amputees in the very first take-home trial (Hargrove et al., 2018). There are supplementary control strategies that can help address the shortcomings of pattern recognition. Collecting data on joint position (Adewuyi et al., 2017) and reducing classification errors made by variations in muscle contraction and mobility (Samuel et al., 2019) can help improve performance. Though these additional control strategies can provide simultaneous control and enhance overall use (Young et al., 2013, 2014; Ortiz-Catalan et al., 2014), they require complex algorithms and rigorous training for high performance achievement.

#### Regression Based Algorithms

A potential solution for achieving simultaneous and proportional is regression-based control. This algorithm allows for classification of multiple movements at one time through estimation of different EMG signals. This provides intuitive control of multiple DoFs at the same time (Smith et al., 2015; Wendelken et al., 2017; Hahne et al., 2018). For example, a person could extend their elbow and rotate their wrist simultaneously. In comparison to conventional control methods or pattern recognition, regression-based methods have demonstrated improved performance with independent control of movement velocity and a more natural feeling (Kuiken et al., 2016; Hahne et al., 2017, 2018). Although this type of control has promising potential, similar to pattern recognition, the differences between the training environment and real-life make it challenging for use outside the laboratory setting (Jiang et al., 2009). Progress is slowly being made with an 8-week at home trial demonstrating successful application in everyday life with regression performance exceeding conventional control (Hahne et al., 2020). More longitudinal studies are needed to demonstrate stability of performance over time.

### ENG Signal Enhancement and Processing

Similar to EMG based control methods, ENG signals can also be analyzed for the application of neurprostheses. These methods address the challenges posed by EMG control including muscle fatigue, stimulation of surrounding tissues, and desensitization of cells. Direct interfacing with neural electrodes can help with specificity of signal but face their own obstacles with biocompatibility and tissue damage related to their level invasiveness. The best physical interface will balance the electrical properties of the system to isolate different sensory precepts, while maintaining a safe and stable environment. Techniques have been employed to improve usability of neural electrodes through changes in electrode configuration, electrical connectors, biocompatibility, and surgical techniques to not only enhance longevity but also facilitate signal extraction and processing.

#### Recording

ENG signals are recorded from peripheral nerves differently depending on the invasiveness of the electrode that is used. Extraneural electrodes, such as the cuff, record a population of signals with information from the overall nerve, whereas intraneural electrodes penetrate the nerve and record spike activity from individual axons (Hong et al., 2018). The raw ENG signals have low signal to noise ratio and are impacted by interference from surrounding muscles, micromotion, and neighboring axons (Tam et al., 2019). Thus, pre-processing with wavelet denoising and different types of filters are necessary prior to channel selection (Micera et al., 2011). This step in processing is complex and expensive, requiring dimensionality reduction algorithms or component analysis with significant computational power.

When population activity recordings are obtained, as in the case of extraneural electrodes, both motor and sensory activity is included and thus algorithms must be used to separate the sources so different features can be extracted. In the case of intraneural electrodes neural spikes are recorded and can be processed using spike sorting algorithms (Cracchiolo et al., 2020). Different axons will generate unique spike activity that is associated with a distinct motor activity. Once the sorted spike signals and population activity features are extracted, they can be used with classification algorithms for prediction of movement intention (Raspopovic et al., 2014). The details of these classification algorithms are beyond the scope of this review.

In humans there have been several successful demonstrations of ENG signal decoding and classification of hand movements. The spike denoising and sorting algorithms as described above have been used with tLIFEs (Micera and Navarro, 2009; Micera et al., 2011) and USEAs (Davis et al., 2016). More recently multi-class neural motor decoding with TIME allowed for velocity and force predictions for 11 different grasping positions (Cracchiolo et al., 2021).

Though ENG recording and processing can improve the specificity of signals, there are inherent limitations. The immune response to the implanted electrodes promotes fibrosis over time, eventually leading to electrical impedance and difficulty in recording signals (Raspopovic et al., 2020). In terms of stimulation this can be overcome with increased injection of charge (de la Oliva et al., 2018), however, this does not improve the ability to record motor signals in the peripheral nerves.

#### Stimulation

Restoration of sensory feedback and stimulation of peripheral nerves is inherently challenging given the complexity of the somatosensory system. Sensory axons outnumber motor axons by at least 10:1. Unlike with motor control where a constant firing can produce a movement, stimulation for sensation requires an interpretation of signals originating from a multitude of different receptors. It is the differential firing between these fibers that elicits the sensation of natural touch. A constant firing instead will generate a very unnatural sensation (Dhillon and Horch, 2005). In terms of translating natural sensation in a prosthetic device, the sensations stimulated must resemble the spatial and temporal characteristics of an intact limb (Horch et al., 2011; Raspopovic et al., 2014; Tan et al., 2015). Neural modulation using pulse shape and frequency can determine quality and intensity of the sensation that is being perceived, respectively (Tyler, 2015; Gracyzk et al., 2016; Valle et al., 2018). Biomemetic encoding strategies can generate optimal stimulation patterns that can effectively produce natural sensation, reduce phantom limb pain, and improve prosthetic embodiment (Raspopovic et al., 2021).

Although signal processing may be easier with stimulation, compared to motor recording, there are concerns regarding safety of electrical stimulation. This involves biostability and consideration of tissue response, as well as identification of acceptable stimulation parameters. Specifically with stimulation of sensation there are numerous different peripheral nerve fibers each with a unique ability to withstand electrical stimulation (Johansson and Flanagan, 2009). Extraneural electrodes currently demonstrate superior stability compared to intraneural electrodes given the level of invasiveness (Günter et al., 2019). However, more work must be done to classify neuronal damage and identify the limits of stimulation parameters to ensure long term safety.

Electrodes must be designed in a way that takes advantage of electrical characteristics using computational modeling for optimization of contacts and subsequent isolation of specific sensory qualities without damaging the surrounding tissue. This allows for processing and interpretation of sensory information in a way that makes the sensation feel like an intact limb. Furthermore, this sensory information closes the loop and provides accurate output information that can be used for advancing motor decoding algorithms.

## Prosthetic Technology

All of the electrodes, surgical techniques, and control methods discussed thus far have been designed to create an interface capable of high-fidelity prosthetic control. These methods have focused on manufacturing a biocompatible, closed looped system with the ability to transmit bidirectional signals for prosthetic control. While these developments have improved functionality, they do not address all of the problems associated with usability. This is influenced by the prosthetic device itself. Device characteristics such as availability, capability, fit of the socket, durability, and cost are all instrumental in implementing daily use of an advanced prosthesis (Samuel et al., 2019). The following section investigates the barriers to clinical translation and subsequent take-home use of the different interfaces.

### Fully Implantable System

In order to implement this type of prosthetic system, it must be fully implantable. In other words, there can be no external wires protruding through the skin for electrical connections. This type of wiring is prone to breakage, infection, and thus is limited to the lab setting. While more invasive electrodes can improve signal transmission, it requires percutaneous wired electrodes. Solutions to combatting this problem include wireless signal transmission or use of an osseointegrated implant. Creating a reliable, wireless communication system with preserved signal strength is a challenging endeavor. It requires the use of appropriately packaged electronics capable of efficient and safe power management in a way that does not require larger or more implantable hardware (Borton et al., 2013; Seo et al., 2016). The most successful wireless design thus far is the IMES system, which uses implantable sensors within the residual limb for prosthetic control (Weir et al., 2009). It is currently being used in clinical trials and has demonstrated effective and reliable signaling in TMR patients (Merrill et al., 2011; Pasquina et al., 2015). Other potential designs explore the use of magnetic beads (Herr et al., 2020) and implantable capsules. In rodents, implantable capsules have demonstrated reliable and robust signal transmission using small electronics and inductive battery power (Deshmukh et al., 2020).

Osseointegration offers a solution for the external, percutaneous wiring that does not require wireless communication. The implant uses an abutment fixated within the bone that can be used to house the wired electrical connectors (Ortiz-Catalan et al., 2014). Although the OI implant is percutaneous, the electrical wiring housed within is protected from the problems associated with the external environment.

Successfully integrating a fully implantable system is the first step in creating a device that is ready for home and clinical use. For this to be applicable for long-term use, other considerations must be taken into account such as the feasibility of hardware implantation and/or removal (if necessary), as well as the long-term safety of bioelectronics.

### Real Time Performance vs. Offline Performance

In addition to physical connections and environmental interference, there are various requirements for signal processing that complicate use of myoelectric prostheses outside of the lab. The laboratory environment represents a controlled, predictable setting that is far different from the dynamic home environment. Conditions within the lab do not simulate real life situations and cannot account for unpredictable, changing movements associated with activities of daily living. The lack of dependability and the potential for unwanted movements creates a dangerous situation that may harm the individual or damage the prosthetic device. While performance in the offline setting demonstrates successful outcomes, it is measured using passive data collection and classification accuracy– the ability of the algorithm to identify certain movements (Nilsson et al., 2017). Though the classification accuracy may be high, the completion rate of tasks can be up to 30% lower (Li et al., 2010). Thus, classification accuracy does not necessarily correlate with functional outcomes in real time control and is not a sufficient comparison tool for control methods. Instead, it is essential that online performance testing be used for a more accurate assessment (Jiang et al., 2009; Ortiz-Catalan et al., 2014; Hargrove et al., 2018). This involves additional interpretation of data during active movement for improved real-time performance (Woodward and Hargrove, 2019). When practiced over a long period time, the EMG patterns become more easily repeatable and adaptable with sustained high-quality performance (He et al., 2015).

The metrics to evaluate performance are often arbitrary, making it challenging to compare outcomes between studies (Tabor et al., 2018). Without standardized criteria, it is difficult to objectively state how “selective” a particular interface is. In order to draw meaningful conclusions, comparative studies with standardized outcome measurements are needed. This involves a systematic evaluation throughout training—assessing progress, functional outcomes, and quality of life (Balk et al., 2019). Until more standardized assessments are implemented, it will be difficult to accurately predict function outside of the lab.

### Training Systems

Despite the increasing sophistication of machine learning algorithms and artificial intelligence, user training remains a critical component of functional success. Standard rehabilitation programs are rigorous and focus on regaining muscle strength and coordination. The tasks are both mentally and physically exhausting, requiring long-term commitments. This process can become especially challenging when an individual no longer has a coach or a therapist to provide motivation (Prahm et al., 2017). Many of the conventional training methods are laboratory-based and lack the mental stimulation required to keep users engaged. To increase motivation and participation, alternative training systems with a game-based model have been developed. These include computer gaming, virtual reality environments, and augmented reality (Resnik et al., 2011; Winslow et al., 2018; Boschmann et al., 2021). Several studies have demonstrated successful prosthesis control with these training systems, in addition to individuals reporting increased usability and motivation (Tabor et al., 2018; Kristoffersen et al., 2021). Not only do these methods improve participant engagement, but they also provide an at-home training system that is more convenient and affordable than daily sessions with a physiotherapist.

Additionally, these training programs help address the remapping of the somatosensory cortex in individuals postamputation. These changes in cortical organization and structural morphology are thought to contribute to phantom limb pain and impact functional ability, hindering prosthetic use (Zhang et al., 2018). While motor cortex may remap, the sensory cortex appears to remain more fixed throughout adulthood with studies showing visuo-tactile mismatches that do not resolve (Ortiz-Catalan et al., 2020). However, numerous studies have demonstrated that prosthetic rehabilitation programs can positively impact neural plasticity with a reduction in phantom limb pain and improved function (Preißler et al., 2017). This has been seen in upper limb amputees with improved grasping and manipulation of objects (Cuberovic et al., 2019) and in lower limb amputees with improved posture control and balance (Dietrich et al., 2018; Bramati et al., 2019).

While these alternative training methods demonstrate functional outcomes like conventional methods, they address a key cause of prosthetic abandonment: lack of motivation and an unwillingness to commit to a rehabilitation program. Despite these challenges, it is necessary to activate mechanisms of the brain that contribute to changes in somatosensory plasticity, a critical component of nerve recovery.

### Commercial Devices

While innovative surgical techniques and complex control methods continue to expand possibilities for amputees, function is largely dictated by the actual prosthetic device. The decision to pursue a bionic limb is mainly determined by affordability and availability. Upper limb devices have become increasingly sophisticated with several companies designing different devices and producing prosthetic components. Though these bionic hands have yet to achieve the same dexterity as the natural fingers and thumb, they are capable of completing activities of daily living. The cost of these below the elbow devices range anywhere from 8,000 to 60,000 USD. Above the elbow options are even less affordable, costing a minimum 50,000 to upwards of 100,000 USD. There are less market options for bionic limbs of the lower extremity, however, the devices that are available are similar in cost to those of the upper extremity. Unfortunately, given the limited availability and affordability of many of these devices, individuals may find themselves needing to purchase different components from different companies to complete their prosthesis. Additionally, there is a lack of communication between the stakeholders in device design and prosthesis control. In an effort to mitigate these problems and improve collaboration within the field, the University of Michigan has developed an open-source initiative for bionic limbs (https://opensourceleg.com). A more viable prosthetic solution capable of supplying the market's needs would improve availability and help with cost reduction as competitors adopt it.

### Prosthetic Embodiment/Sensation

Overall use of a prosthesis is not only dependent on availability or operability, but most importantly, on an individual's desires and goals. This may be related to occupation or hobbies, or to their level of function before the amputation. It may even be influenced by a patient's motivation and dedication to the training required to use one of the advanced prosthesis (Kerver et al., 2020). Furthermore, co-morbidities and health status may limit an individual's ability to explore more invasive options. Whatever the case, it is likely that the primary objective will vary between individuals. Thus, the conversation regarding goals is an essential one that must take place early so that expectations can be established and prosthesis use can be optimized.

While comparative studies and long-term effectiveness of prostheses remain limited, there is research exploring prosthesis abandonment and the qualities that are most important to the individuals who are using them. A survey study by Zheng et al. found that improved dexterity, durability, and sensation were the most important qualities for amputees (Zheng et al., 2019). Similarly, a case series with 3 female amputees found advanced prostheses to be desirable if they increased abilities and if device support was available (Resnik et al., 2019). The most common concerns regarding these devices include cost, durability, invasiveness of surgery, and ease of use (Engdahl et al., 2015, 2017; Resnik et al., 2017). Though many of these studies demonstrate shared desires, they do not necessarily indicate shared experiences or lifestyles.

Improvement of sensation is a critical area of ongoing research in the field of prosthetics. Numerous studies have demonstrated the value of sensation in improving control, adaptability, and embodiment (Sinha et al., 2011; Schiefer et al., 2016, 2018). Prosthesis abandonment rates range anywhere from 24 to 44%, with lack of sensation being a significant contributing factor (Biddiss and Chau, 2007; Salminger et al., 2019). When an individual is unable to feel their prosthetic limb, it does not feel like a part of their body or self and discourages overall use. This lack of sensation can put both the user and the device at risk for harm and damage. Additionally, when sensation is restored, there are a number of psychosocial factors that improve including social interaction, sense of self, and overall quality of life (Graczyk et al., 2018; Page et al., 2018; Petrini et al., 2019a; Middleton and Ortiz-Catalan, 2020).

After a type of prosthesis is decided upon, rehabilitation and incorporation of the device must occur. This process involves a large multidisciplinary team that extends beyond the surgeons and engineers who helped create the device. Wound care specialists and medical management are important for ensuring sufficient recovery and appropriate fitting of a prosthesis. Occupational therapists, physical therapists, and prosthetists help prepare an individual for prosthesis training (Cancio et al., 2019). Early rehabilitation and training are not only necessary for maximizing functionality, but are also associated with improved patient satisfaction (Resnik et al., 2020). This training can occur long before an individual is even fitted for a prosthesis.

## Discussion

During the last few decades, considerable strides have been made in the field of neuroprosthetics. With advanced robotic technology and revolutionary surgical techniques, the use of a fully integrated, bidirectional prosthesis is now within the realm of possibility for amputees. Though the technology is available to make these improvements, there is division within the industry that makes it challenging to take technical ideas and translate them into the commercial world. One of the most significant limiting factors for this translation is the inability for one PNI to carry out all the functions required to fully operate an advanced neuroprosthetic. Individually, the existing interfaces have significantly advanced or improved a particular aspect of the bidirectional system (Table 1). For example, extraneural electrodes have demonstrated chronic long-term stability, while intraneural electrodes offer a promising solution for high-fidelity signaling. Additionally, neural electrodes have shown their ability to restore sensory feedback and subsequently enhance motor control with improved object manipulation and force control (Tan et al., 2014; Oddo et al., 2016). This allows for precise stimulation capable of eliciting natural-like sensations while balancing specificity and cognitive load. Repetitive motor input with visual feedback alone is not capable of adjusting sensory cortical maps (Ortiz-Catalan et al., 2020). Although direct stimulation of the nervous system can successfully restore tactile sensations, proprioceptive precepts are much more challenging.

**Table 1:** Characteristics of the electrodes and surgical techniques.

**Electrodes**	**Longevity**	**Motor**	**Sensation**	**Pain**	**Upper/and or Lower Limb**	**Technological capability**
Cuff	11 years	Improvement in hand grip	-Grasping and slippage control; manipulation of objects -Improved postural stability	Elimination of PLP that persists in the absence of stimulation	UL and LL	Closed loop control in humans
FINE	3 years	Improved balance and mobility	-Grasping and slippage control	Reduced PLP	UL and LL	Closed loop control in humans
LIFE	3 months	Improvement in force control	Grasping and slippage control; manipulation of objects	Reduced PLP	UL	Closed loop control in humans
TIME	6 months	-Improvement in force control -Improved coordination and dexterity	Recognition of texture, shape, and size of objects	Decreased PLP when sensory feedback provided	UL and LL	Closed loop control in humans
USEA	14 months	-Independent control of 5 DoF -Intuitive and dexterous control	-Coordination grasp and sensory responses -Perception of at least 131 sensory precepts	Reduced PLP	UL	Closed loop control in humans
Regenerative	N/A	N/A	N/A	N/A	N/A	Animal models
**Surgical techniques**	**Longevity**	**Motor**	**Sensation**	**Pain**	**Upper/and or Lower Limb**	**Technological capability**
TMR		-Increased mobility, balance and confidence while walking	Sensations elicited with TSR; variable results, unnatural feeling	Reduction in neuroma formation, neuroma pain, PLP pain, and opioid use	UL only	Closed loop control in humans (take home)
RPNI	10 months	-Fine motor control of intrinsic hand muscles (control of one and two DoF finger)	N/A	Reduction in neuroma formation, neuroma pain, PLP pain, and opioid use	UL only	Open loop in humans (no sensory component)
AMI	24 months	-Capable of producing high fidelity efferent signals, reflex arcs -Prevents disuse atrophy	-Restoration of proprioception -Increased prosthetic embodiment	Reduction in phantom sensations and cutaneous pain likely related to concurrent TMR and RPNI	LL only	Closed loop in humans
ONI	N/A	N/A	N/A	N/A	N/A	Animal models

While many existing surgical techniques have not been as successful in providing sensory feedback, they have demonstrated sophisticated motor capabilities. TMR provides movement for missing muscles with multiple DoF, whereas RPNI has demonstrated motor control selective enough for movement of intrinsic hand muscles. Osseointegration allows for better mechanical stability and increased ROM, and ONI has the potential to provide high fidelity signaling through the use of regenerative electrodes within the medullary canal. Additionally, all of these surgical techniques are a treatment option for postamputation pain and symptomatic neuromas. Although the AMI does not directly improve motor control or treat neuromas, it is the first PNI to restore proprioceptive feedback. Summation of the advantages and disadvantages of the electrodes and surgical techniques are outlined in Table 2.

**Table 2:** Advantages and disadvantages for prosthetic application.

**Electrodes**	**Advantages**	**Disadvantages**
Cuff	-Stability and Longevity -Discrimination among sensory percepts of different qualities at different locations -Eliminates PLP even in the absence of stimulation -Upper and Lower limb capabilities	-Limited specificity of signals -Difficulty with recording for motor control -Requires higher level stimulation
FINE	-Stability and Longevity -Discrimination among sensory percepts of different qualities at different locations -Reduced PLP with stimulation -Improvement in force control -Upper and Lower limb capabilities	-Limited specificity of signals -Difficulty with recording for motor -Requires higher level stimulation
LIFE	-Sensory feedback improves grasping performance and manipulation of objects -Proximity to nerves allow for low level charge stimulation -Reduced PLP with stimulation -Improvement in force control -Can record higher amplitude signals compared to extraneural electrodes	-Motor recordings challenging over time -Requires a meticulous surgical implantation -Currently only in Upper limbs
TIME	-Improved force control, motor coordination and dexterity -Discrimination among sensory percepts of different qualities at different locations -Proximity to nerves allow for low level charge stimulation -Reduced PLP with stimulation -Can record higher amplitude signals compared to extraneural electrodes -Upper and Lower limb capabilities	-Concern for longevity -Motor recordings challenging over time -Requires a meticulous surgical implantation -Limited sensory perceptions essential for walking- limb position, torque, and proprioception
USEA	-Most selective neural electrode with potential to provide complex control -Discrimination of sensory percepts by location, quality, and intensity -Proximity to nerves allow for low level charge stimulation -Reduced PLP -Can record higher amplitude signals compared to extraneural electrodes	-Risk for damage; concern for longevity and long-term stability -Requires a meticulous surgical implantation -Motor recordings challenging over time
Regenerative	-Most promising electrode in terms of specificity -Potential treatment for postamputation neuromas	-Not yet used in humans given the concern for safety and damage
**Surgical techniques**	**Advantages**	**Disadvantages**
TMR	-Prevention and treatment for postamputation pain -Available for take home use -does not require unnatural code switching -does not require intact distal muscles -has demonstrated improved control with pattern recognition -can be performed at the time of amputation surgery or later as a second surgery -TSR is a potential solution for bidirectional feedback	-Currently only for upper limb -Limited by EMG cross talk from surface electrodes -Number of control sites is limited by availability of denervated, target muscles -Thus far results with TSR have been variable with many of the elicited sensations feeling unnatural; also requires large surface area
RPNI	-Prevention and Treatment for postamuptation pain -Fine motor control (individual finger movements) -Implantable electrodes reduce EMG cross talk -Use of free muscle grafts; not limited by availability of residual muscle	-Currently only for upper limb -Limited by percutaneous wiring, not available for take home use -No published data on sensation
AMI	-Can restore natural proprioception -Closed loop joint torque control -Prosthetic Embodiment -Can be combined with other interfaces to improve motor control, sensation, and reduction of pain	-Complex surgery -Currently only for lower limb -Lacks fine motor control (will be more difficult in upper limb prosthetics) -does not directly treat postamputation pain
ONI	-Prevention and Treatment for postamputation pain -Mechanical stimulation and Osseoperception -Prosthetic embodiment -Superior to socket prosthesis with Increased ROM and mechanical stability -Medullary cavity provides space for implantable biotechnology -Can be implemented as a primary surgery or secondary surgery	-Not yet evaluated in humans -Concern for infection and implant failure with osseointegration

Despite all the progress that has occurred in specific areas of the field, no single PNI can perform the needed functions all on its own. The path forward requires a comprehensive approach with collaboration between all stakeholders—surgeons, biomedical engineers, prosthetists, device companies, and most importantly, the patient. Different technologies with unique capabilities should be combined to best suit the patient's needs and optimize quality of life. Until recently, most innovation within the prosthetic field has been created in silos. Although this work has led to substantial progress, without coordination, it can be redundant and inefficient, and only further perpetuates the gap between academia and clinical utility. A collaborative approach encourages facilitation of standardized performance metrics and promotes translation of advanced prosthetics into the commercial, and thus practical world. The technology is available; what is needed is a way to synthesize and coordinate the diverse work.

Fortunately, the field has already begun to see some joint efforts come to fruition. For example, the use of TMR with neural and muscular electrodes in upper extremity amputees allows for bidirectional communication and improved somatosensory control (OrtizCatalan et al., 2020). In addition, use of osseointegration further increases range of motion and prosthetic embodiment (Vincitorio et al., 2020). Combination of TMR and RPNI capitalizes on the advantages of each individual design to optimize efficacy (Valerio et al., 2020). Even new surgical constructs such as the AMI rely on the integration of other PNI designs to provide more comprehensive functions (Herr et al., 2020). Though AMI can restore proprioception, it depends on concurrent use of TMR and/or RPNI for motor control and neuroma treatment. Similarly, the ONI takes advantage of neuronal regeneration and the medullary canal environment to optimize use of regenerative nerve electrodes (Millevolte et al., 2021). Potential combination of different PNIs is presented in Table 3. There are many variations listed, but this table is not exhaustive. While these synergistic combinations demonstrate the advantages of collaboration, they are only the beginning. Currently none of these technologies are suited to meet the broader amputee community's needs. As these technologies extend into clinical trials, as TMR and RPNI have (at least for treating neuroma), they are then going to have to meet all of the regulatory requirements of each country/region if they are to be broadly accepted and clinically applied. As with any medical device, each implantable device required for prosthetic control is going to need to be classified for regulatory purposes, which in the US comes under the jurisdiction of the Food and Drug Administration, as stated in the Code of Federal Regulations for medical devices (Title 21, 800 series).

**Table 3:** Potential Combination of different PNI's.

**Sensation**	**Motor**	**Pain**	**Mechanical Stability**
Extraneural Electrode^∧^	RPNI	RPNI	OI
Extraneural electrode^∧^ + AMI^*^	TMR and/or RPNI	TMR and/or RPNI	OI
AMI^*^	RPNI	RPNI	OI
ONI^∧^ + intraneural electrode^∧^	RPNI	RPNI	OI
ONI^∧^ + intraneural electrode^∧^	RPNI	ONI	OI
ONI^∧^ + intraneural electrode^∧^	TMR	RPNI	OI
Extraneural electrode^∧^	TMR	TMR	OI
TSR^∧^+AMI^*^	TMR	TMR	OI
TSR^∧^+AMI^*^	TMR and/or RPNI	TMR and/or RPNI	OI
ONI^∧^+intraneural electrodes + AMI^*^	TMR and/or RPNI	TMR and/or RPNI	
ONI^∧^+regenerative electrodes^∧^	ONI+regenerative electrodes	ONI	OI
AMI^*^	AMI	RPNI, TMR, or ONI	OI

* *Restores proprioception*.

∧ *Restores sensory modalities other than proprioception*.

Incorporation of this technology will be dependent on the patient, technological availability, and surgical implementation. The potential need for secondary surgery is not yet clear given the infancy of these devices. In the majority of cases, these devices have been implemented in persons already living with amputation, who may be returning to have neuropathic/phantom pain managed, at which point an interface is implanted as part of an already necessary secondary procedure (Di Pino et al., 2014). However, use of the surgical techniques such as TMR and RPNI, have been performed at the time of amputation to prophylactically prevent neuropathic/phantom pain (Santosa et al., 2020). For many involved in prosthetic control studies, patients have been heavily vetted to meet long-term study requirements. As evidenced by the increasing uptake of osseointegration, constant refinements have enabled what is more commonly a two-step surgical procedure to be reduced to a single surgical procedure as the technologies and techniques have become more refined with practice (Hoellwarth et al., 2020). The decision by patients over what is more suitable and more widely accepted is going to be heavily based on what the technology can achieve relative to what the individual desires. This is highlighted by the increased uptake of osseointegration by patients of standard socket prosthesis, given that it is a highly invasive, secondary procedure, increasing numbers of patients are seeking OI globally for its improved functionality and the subsequent improvements in quality of life (Souza et al., 2020). From the perspective of sensory restoration, there is yet to be a technology that does not require implantation of an electrode to provide chronic restoration of sensation.

Given the current state of technology and adaptive surgical strategies, there are theoretically a multitude of unique PNI combinations that can be tailored to an individual's needs in order to maximize function and improve quality of life. In the case of a lower extremity amputee with the desire to return to his love of hiking, TMR or RPNI, AMI, OI, and a FINE electrode could be used to provide sophisticated motor skills, proprioception, increased ROM, and tactile sensory feedback, respectively. If an amputee with a socket prosthesis already underwent TMR surgery but is seeking further functional improvement, the ONI can be performed as a revisionist procedure with installation of an osseointegrated implant for improved usability and use of neural electrodes for sensory feedback. If the primary goal is optimizing prosthetic embodiment, either the AMI or ONI should be used for restoration of proprioception or osseoperception, respectively. When an individual is not interested in using a prosthesis, but is seeking optimal pain control, TMR, RPNI, and ONI are all viable options for prevention or treatment of postamputation pain. These surgical approaches alone provide a wide range of application to all of the currently available interfaces, including those considered most invasive. Different combinations will be more suitable for certain individuals than others. While an ultimate decision relies on availability of technology and surgical accessibility, it is still largely dependent on a patient's desires. Even if there were a single PNI that could “do it all,” it likely would not be suitable for every patient. Thus, the goal should not be directed at developing a universal prosthesis but should instead be aimed at employing creative strategies to encourage collaboration and provide unique options that can be tailored to each individual.

## Conclusion

In this review, we summarize the current landscape of Peripheral Neural Interfaces and the progress that has been made to advance the field of prosthetics. While the technological advancements in prosthetic devices have been remarkable, their use is severely limited by market availability, take home ability, and lack of sensation. Moreover, progress in the field has not been coordinated and this has limited the ability to serve patients' needs and optimize their quality of life. In this comprehensive investigation, we argue for the importance of collaboration and the use of interfaces in combination with each other to offer each patient the best quality of life.

## Author Contributions

AK developed the concept and idea with support and help from other contributing authors, and took the lead in manuscript writing. AD provided support in concept development, manuscript writing, and provided critical feedback in manuscript revision. SP offered support in concept development, manuscript writing, and provided critical feedback in manuscript revision. All authors contributed to the article and approved the submitted version.

## Conflict of Interest

The authors declare that the research was conducted in the absence of any commercial or financial relationships that could be construed as a potential conflict of interest.
